# The Impact of Minimal Residual Disease (MRD) Testing on the Decision-Making Process in Non-Small-Cell Lung Cancer (NSCLC)

**DOI:** 10.3390/cancers18081246

**Published:** 2026-04-14

**Authors:** Roni Gillis, Tamar Zahavi, Nir Peled, Adar Yaacov, Basel Afifi, Jaber Salim, Reham Basheer, Noam Asna, Arnon Makori, Michael Peer, Evgeni Gershman, Yoav Manaster, Osnat Moreh Rahav, Elizabeth Dudnik

**Affiliations:** 1 The Helmsley Cancer Center, The Eisenberg R&D Authority, Shaare Zedek Medical Center, 12 Shmuel Bait St., Jerusalem 9103102, Israel; 2 Faculty of Medicine, The Hebrew University of Jerusalem, Jerusalem 9190500, Israel; 3 ProGenetics, 2 HaMatzuda St., Azor 5800146, Israel; 4 Imaging Services, Assuta Medical Centers, 20 HaBarzel St., Ramat HaHayal, Tel Aviv 6971028, Israel; 5 Department of Thoracic Surgery, Tel Aviv Medical Center, 6 Weizmann St., Tel Aviv 6423906, Israel; 6 Faculty of Medicine, Tel-Aviv University, P.O. Box 39040, Tel Aviv 6997801, Israel; 7 Department of Pulmonology, Kaplan Medical Center, 1 Pasternak St., Rehovot 7661041, Israel; 8 Thoracic Oncology, Assuta Medical Centers, 20 HaBarzel St., Ramat HaHayal, Tel Aviv 6971028, Israel; elizabeth.dudnik1603@gmail.com; 9 Faculty of Health Sciences, Joyce & Irving Goldman Medical School, Ben Gurion University of the Negev, P.O. Box 653, Beer-Sheva 84105, Israel

**Keywords:** minimal residual disease (MRD), circulating tumor DNA (ctDNA), liquid biopsy, lung cancer, clinical impact, treatment de-escalation

## Abstract

**Background**: Minimal residual disease (MRD) assessment is an emerging tool for refining the risk of relapse following definitive therapy in non-small-cell lung cancer (NSCLC). However, data regarding its clinical impact on the decision-making process remain limited. We evaluated MRD feasibility and its impact in the real-world setting. **Methods**: A pooled retrospective analysis of longitudinal MRD data in NSCLC patients (n = 34: Signatera™ (Exome), n = 25, Guardant Reveal™, n = 9) was implemented. Co-primary endpoints: MRD feasibility and clinical impact on management (changes in surveillance intensity or therapy escalation/de-escalation). Secondary endpoints: sensitivity, specificity, positive predictive value (PPV), negative predictive value (NPV), accuracy for recurrence detection, and MRD lead time. **Results**: MRD was feasible in 32/34 patients (94.1%); longitudinal testing included two samples in 15 patients (44.1%) and three samples in 2 patients (5.9%). Signatera™ (Exome) failed in 2/25 (8.0%) due to insufficient tissue. MRD influenced management in 20/34 (58.8%) patients, most commonly supporting therapy de-escalation (15/34, 44.1%), followed by imaging surveillance modification (3/34, 8.8%) and therapy escalation (2/34, 5.9%). In univariable analysis, tumor grade and STAS were associated with MRD-driven management impact, but neither remained significant in multivariable analysis. With a median follow-up of 18.9 months (IQR 8.5–30.7), MRD was positive in 6/32 (18.8%), while recurrence/progression occurred in 10/32 (31.3%) patients. MRD yielded 21 true negatives, five true positives, five false negatives (including two isolated brain recurrences), and one false positive, corresponding to a sensitivity of 50.0%, specificity of 95.5%, PPV of 83.3%, NPV of 80.8%, and an accuracy of 81.3%. The median MRD lead time (n = 5) was 1.31 months (range, 0.46–5.52). **Conclusions**: In this real-world cohort, MRD testing was feasible and frequently guided clinical decisions, mainly supporting treatment de-escalation. MRD was highly specific but less sensitive. Prospective studies are needed to define optimal testing intervals and validate MRD-guided strategies.

## Simple Summary

After cancer treatment, tiny amounts of tumor can remain in the body, known as minimal residual disease (MRD). New blood tests can detect fragments of tumor DNA circulating in the bloodstream, potentially revealing hidden cancer before it becomes visible on scans. In this study, we examined how these blood-based MRD tests were used in the care of 34 patients with lung cancer who were treated at two medical centers in Israel. We found that MRD testing was successfully performed in most patients and influenced treatment decisions in over half of the cases, most often by reassuring doctors that additional treatment could safely be withheld. While the test was very accurate when it detected residual cancer, it missed some cases of recurrence, particularly when cancer recurred in the brain. Our findings suggest MRD testing can help personalize lung cancer care, but further studies are needed before it can reliably guide routine treatment decisions.

## 1. Background

Lung cancer remains the leading cause of cancer-related mortality worldwide, with non-small-cell lung cancer (NSCLC) accounting for approximately 85% of cases. Despite curative-intent surgery for early-stage disease, recurrence rates range from approximately 20% for stage I to over 50% for stage III, driving the use of adjuvant systemic therapy. However, current clinicopathologic staging incompletely identifies patients who will derive meaningful benefit from adjuvant treatment, highlighting the need for refined risk stratification tools [1].

Minimal residual disease (MRD)—the detection of circulating tumor DNA (ctDNA) in plasma after definitive oncologic therapy—captures occult residual malignancy below the sensitivity of standard imaging and marks a clinically meaningful transition between apparent remission and overt relapse. In resected stage I–III NSCLC, postoperative ctDNA positivity at landmark and/or serial time points is consistently associated with markedly inferior recurrence-free survival, and ctDNA detection frequently precedes radiographic recurrence by several months (reported medians ~88–212.5 days), offering a lead time not achievable with routine computer tomography (CT)-based surveillance [2,3,4,5,6,7]. The prognostic value of MRD extends beyond surgical cohorts: in unresectable locally advanced NSCLC treated with definitive chemoradiotherapy (CRT), post-treatment ctDNA detection identifies a high-risk population with substantially inferior outcomes (reported hazard ratios ~5.6–14.8 for detectable vs. undetectable ctDNA), and ctDNA dynamics during CRT and early consolidation further stratify relapse risk in the durvalumab era [7,8,9]. These data are increasingly relevant as adjuvant and consolidation options expand, while conventional clinicopathologic selection remains imperfect. Multiple cohorts suggest that ctDNA may refine postoperative risk and even predict adjuvant chemotherapy benefit, supporting an MRD-informed framework for both escalation in high-risk patients and potential de-escalation in those with persistently undetectable ctDNA across serial assessments [10,11,12].

MRD assays in NSCLC are broadly categorized as tumor-informed and tumor-agnostic approaches [13]. Tumor-informed platforms (e.g., Signatera™ (Exome), Natera) personalize variant tracking using tumor tissue and then longitudinally interrogate those patient-specific variants in plasma, typically achieving very high specificity/positive predictive value (PPV) because detection is anchored to multiple tumor-derived mutations and is less susceptible to non-tumor noise [5,13]. Tumor-agnostic, tissue-free assays (e.g., Guardant Reveal™, Guardant Health) rely on fixed plasma panels and may incorporate multiomic signals (e.g., methylation/fragmentomics), enabling faster deployment when tissue is limited; performance is also advancing with ultrasensitive and machine-learning-guided signal enrichment methods [13,14,15]. Despite compelling prognostic validity, key implementation gaps persist, such as sensitivity at early landmark time points remains modest across studies (clinically meaningful false negatives), isolated central nervous system (CNS) relapse may be under-detected due to limited ctDNA shedding, and optimal sampling frequency/duration are not standardized [13,16,17,18]. Most importantly, evidence for clinical utility is still limited—real-world data remain sparse on how MRD results actually alter surveillance intensity or drive treatment escalation/de-escalation across heterogeneous NSCLC populations and assay types, and whether those MRD-driven decisions align with subsequent outcomes [11,12,13].

Understanding how MRD testing is currently deployed in routine practice and how results influence clinical decision-making is a critical translational step. We therefore conducted a two-center retrospective analysis of NSCLC patients undergoing MRD testing to evaluate feasibility, characterize MRD-driven modifications in management (including treatment escalation, de-escalation, and surveillance changes), and assess concordance between MRD results and radiologic relapse, including molecular lead time where applicable.

## 2. Methods

### 2.1. Study Design

We conducted a retrospective, pooled analysis of consecutive patients with NSCLC who underwent longitudinal ctDNA-based MRD testing during routine clinical care at two oncology centers. This study was designed to evaluate the real-world feasibility of MRD testing and the impact of MRD results on the clinical decision-making process. Data were extracted from institutional medical records and included clinicopathologic characteristics, treatment details, MRD testing results and timing, imaging surveillance, and recurrence outcomes.

This was an exploratory, hypothesis-generating study; no formal sample size calculation was performed. The cohort comprises all consecutive patients who underwent MRD testing at the two participating centers between October 2019 and May 2025.

### 2.2. Patient Population

Eligible patients had histologically confirmed NSCLC with at least one MRD test ordered following receipt of oncological treatment (either local, e.g., surgery/definitive radiotherapy, or systemic treatment). Patients were included irrespective of disease stage or treatment type. Patients without an evaluable MRD result (e.g., assay failure) were included in feasibility and clinical impact analyses but excluded from MRD performance and concordance analyses.

### 2.3. MRD Assessment and Definitions

MRD was assessed using commercially available ctDNA assays selected at the physician’s discretion, including a tumor-informed personalized assay (Signatera™ (Exome), Natera, Austin, TX, USA) and a tumor-agnostic tissue-free assay (Guardant Reveal™, Guardant Health, Palo Alto, CA, USA). Longitudinal testing was performed at physician-determined time-points without a standardized sampling protocol. Up to three serial post-treatment MRD assessments per patient were captured and denoted MRD1 (first post-treatment draw), MRD2 (second draw), and MRD3 (third draw), when available.

MRD status was defined dichotomously for each time point: MRD-positive if ctDNA was detected and MRD-negative if ctDNA was not detected. MRD test feasibility was defined as the proportion of patients in whom an interpretable MRD result was obtained.

### 2.4. Surveillance and Clinical Outcomes

Patients were followed with routine imaging per standard clinical practice, with additional imaging as clinically indicated. Specifically, surveillance imaging was performed per institutional protocols consistent with NCCN guidelines, typically including chest CT every 3–6 months; brain MRI and FDG-PET-CT were performed as clinically indicated. Recurrence/disease progression was defined radiologically, and sites of recurrence were recorded, including identification of isolated CNS relapse. The date of recurrence/disease progression was defined as the date of the imaging study documenting disease relapse or progression.

### 2.5. Endpoints

The co-primary endpoints were:

1. The feasibility of MRD testing, defined as the proportion of patients with at least one interpretable MRD result among all patients for whom MRD testing was ordered.
2. The impact of MRD on the clinical decision-making process, defined as any MRD-associated modification to the planned management pathway.

MRD’s impact on management was categorized as one or more of the following:

- Treatment: de-escalation: omission of systemic therapy that would otherwise have been administered based on guideline-concordant clinicopathologic criteria.
- Treatment escalation: initiation of systemic therapy not initially planned (i.e., given despite an absence of conventional indications), attributed to MRD positivity.
- Surveillance modification: intensified radiologic surveillance (e.g., imaging at shorter intervals than planned, or an introduction of more sensitive imaging modalities otherwise not indicated, such as FDG-PET-CT (fluorodeoxyglucose positron emission tomography–computed tomography) or brain MRI (magnetic resonance imaging) triggered by MRD findings.

MRD-driven management impact was ascertained through a retrospective chart review. A management decision was classified as MRD-driven if: (1) the treating physician explicitly documented that the MRD result informed the management change, or (2) the management deviated from guideline-concordant recommendations in a direction consistent with the MRD result (e.g., omission of adjuvant therapy in a patient meeting standard criteria for adjuvant treatment, coinciding with MRD-negative status). All treatment decisions were made at the discretion of the treating oncologist within the framework of shared decision-making.

Secondary endpoint:

- MRD performance for recurrence/progression prediction, reported as sensitivity, specificity, PPV, negative predictive value (NPV), and overall accuracy.

### 2.6. Statistical Analysis

Descriptive statistics were used to summarize baseline patient, tumor, and treatment characteristics and MRD testing patterns. Continuous variables were reported as the median (range), and categorical variables as counts and percentages. Concordance between MRD and radiologic recurrence/progression was assessed using predefined categories (true positive, true negative, false positive, false negative), from which sensitivity, specificity, positive predictive value, negative predictive value, and overall accuracy were calculated.

MRD lead time was defined, among true-positive cases, as the interval between the earliest MRD-positive blood draw and the date of radiologic recurrence/progression, and was reported as the median (range). Analyses were descriptive; no formal hypothesis testing was prespecified, given the limited sample size and retrospective design. To explore factors that were associated with a positive MRD-driven impact on management, univariable and multivariable regression models were built. In the multivariable model, age at diagnosis, gender, tumor grade, and spread through air spaces (STAS) were assessed; effect estimates were presented as adjusted ratios with 95% confidence intervals and two-sided *p*-values (Fisher’s exact test for categorical factors; logistic regression for age). Analyses were performed using a complete-case approach, with statistical significance defined as *p* < 0.05. Analyses were conducted in Python 3.11.2. using AI-assisted tools (ChatGPT version 5.2, OpenAI; Claude version 4.2, Anthropic).

## 3. Results

### 3.1. Patient Cohort and MRD Testing Overview

The pooled cohort comprised 34 patients with NSCLC who underwent ctDNA-based MRD testing as a part of routine clinical management (Table 1). MRD assessment was performed using a tumor-informed assay (Signatera™ (Exome), n = 25) or a tumor-agnostic assay (Guardant Reveal™, n = 9), selected at the clinician’s discretion.

### 3.2. MRD Feasibility

MRD testing was feasible in 32 of 34 patients (94.1%). Of the twenty-five patients referred for MRD testing with Signatera™ (Exome), two patients (8.0%) had no evaluable MRD result due to failure of the tumor-informed assay (insufficient tumor DNA for assay development). Up to three serial post-treatment MRD assessments were available per patient (MRD1-MRD3), with sampling intervals determined by the treating physicians; longitudinal testing included two samples in fifteen patients (44.1%) and three samples in two patients (5.9%). The median interval from treatment to MRD1 was 3.0 months (range, 0.5–33.4; IQR, 2.0–6.4), and the median interval from MRD1 to MRD2 was 4.0 months (range, 1.5–11.7; IQR, 3.4–5.1).

### 3.3. Impact of MRD on Clinical Decision-Making Process

The MRD results influenced clinical management in 20/34 cases (58.8%). Of those, a positive impact of MRD testing was observed in 14/25 cases (56.0%) with Signatera™ (Exome) and in 6/9 cases (66.7%) with Guardant Reveal™. The distribution of MRD-driven interventions is summarized in Table 2. The most common MRD-driven change was therapy de-escalation (15/34 cases, 44.1%) and specifically, omission of adjuvant therapy in MRD-negative patients who would otherwise have been candidates for adjuvant treatment based on clinicopathologic risk assessment (10/34 cases, 29.4%). In four patients with advanced disease under control with effective systemic therapy (4/34 cases, 11.8%), the decision to implement a stop-and-go strategy for otherwise continuous systemic treatment was made based on MRD-negative results. In one additional patient with advanced NSCLC harboring an epidermal growth factor receptor *(EGFR)* exon 19 del mutation (1/34 cases, 2.94%), MRD negativity supported the decision to forgo systemic treatment escalation (e.g., amivantamab plus lazertinib) and to continue osimertinib monotherapy. Less frequent changes included the addition of adjuvant therapy, prompted by MRD positivity despite an initial plan not to administer adjuvant treatment (2/34 cases, 5.9%), and intensification of radiologic surveillance (introduction of FDG-PET-CT and/or brain MRI imaging, otherwise not indicated) triggered by MRD positivity (3/34 cases, 8.8%). In the remaining 14 patients, the MRD results were concordant with the pre-existing management plan and did not trigger a change; the most common scenario was early-stage patients in whom adjuvant therapy was not indicated, regardless of MRD status, or those in whom adjuvant treatment was already planned, irrespective of the MRD findings.

### 3.4. Concordance Between MRD and Radiologic Disease Recurrence/Disease Progression

With a median follow-up since the NSCLC diagnosis of 18.9 months (interquartile range (IQR) 8.5–30.7) and a median follow-up since the MRD1 collection of 9.0 months (IQR 4.3–17.9), among 32 evaluable patients, radiologic recurrence/disease progression following the MRD blood draw occurred in 10 patients (31.3%), whereas MRD positivity was detected in 6 patients (18.8%).

Using MRD status to classify recurrence outcomes yielded five true positives, twenty-one true negatives, five false negatives, and one false positive (Table 3). Two of the five false-negative cases involved isolated CNS recurrence. Importantly, the false-positive MRD designation in a patient with early-stage NSCLC harboring an anaplastic lymphoma kinase *(ALK)* rearrangement is uncertain. MRD1, obtained immediately after adjuvant chemotherapy, was positive and led to the initiation of alectinib; however, a subsequent MRD2 performed while on alectinib was negative, likely reflecting a rapid treatment effect. No radiologic recurrence was documented, which may likewise be attributable to alectinib.

**Figure 1:**
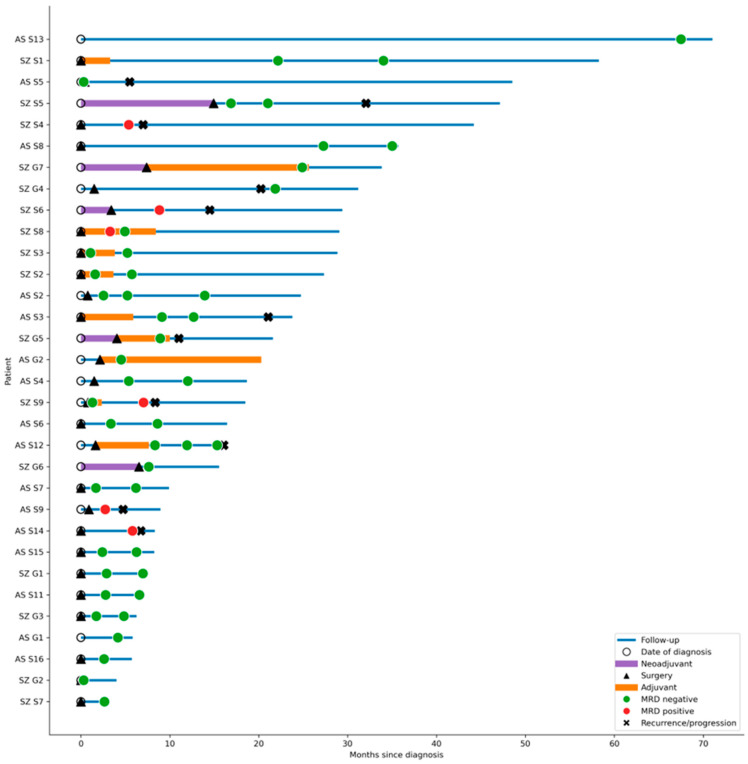
Swimmer plot of longitudinal MRD testing, treatments, and outcomes (n = 32). Each horizontal bar represents an individual patient, with time shown on the *x*-axis as months from initial diagnosis. White circles denote the date of diagnosis, black triangles mark the date of surgery, violet segments indicate neoadjuvant therapy, and orange segments indicate adjuvant therapy. MRD results are shown as green circles (MRD-negative) and red circles (MRD-positive), black “X” denotes radiologic recurrence or disease progression. Bars extend to the last follow-up. Abbreviations: AS—Assuta Medical Centers; G—Guardant Reveal™, Guardant Health; S—Signatera™ (Exome), Natera; SZ—Shaare Zedek Medical Center.

To integrate perioperative treatments, MRD longitudinal testing results, and outcomes, we generated a swimmer plot summarizing individual timelines (Figure 1). Importantly, several patients—particularly those who did not receive adjuvant therapy following an MRD-negative result—remained disease-free during prolonged follow-up.

Corresponding performance characteristics for MRD were a sensitivity of 50.0%, a specificity of 95.5%, a PPV of 83.3%, an NPV of 80.8%, and an accuracy of 81.3% (Table 3).

### 3.5. MRD Lead Time

Among the five true-positive cases with non-negative lead-time values, MRD preceded the radiologic recurrence by a median of 1.3 months (range, 0.5–5.5 months).

### 3.6. Impact of MRD on Clinical Decision-Making Process: Uni- and Multivariable Regression Models

To identify factors that were associated with an MRD-driven impact on management, we constructed univariable and multivariable regression models. In the univariable analyses, tumor grade and the presence of STAS were the only variables significantly associated with a positive MRD-driven impact. However, these associations were not retained in the multivariable model (Table 4).

## 4. Discussion

In this real-world retrospective cohort, ctDNA-based MRD testing was readily implementable in routine care and often informed management decisions in both resected and metastatic NSCLC. Both landmark and longitudinal MRD assessments demonstrated high specificity and PPV for subsequent radiologic recurrence, supporting MRD positivity as a clinically actionable indicator of relapse risk [3,4,5,10,14,15,16,17,18]. In contrast, MRD negativity generally correlated with favorable outcomes, although its interpretation was constrained by clinically meaningful false-negative cases.

A key finding was the substantial real-world impact of MRD on clinical decision-making. The MRD results altered management in 58.8% of patients, most commonly through treatment de-escalation. Specifically, adjuvant systemic therapy was withheld in MRD-negative patients who would otherwise have qualified for postoperative treatment based on standard clinicopathologic criteria. Notably, all but one of these patients remain recurrence-free to date. This signal aligns with prospective data suggesting that MRD-negative patients derive limited benefit from adjuvant chemotherapy, whereas MRD-positive patients may be the population most likely to benefit from systemic therapy escalation [10,11,12]. Having said that, we would like to emphasize that these preliminary findings are hypothesis-generating and should not be used to justify withholding guideline-concordant adjuvant therapy outside of clinical trials. Additionally, MRD negativity supported a stop-and-go approach in a subset of patients with radiographically controlled advanced NSCLC; this is in contrast to otherwise continuous systemic therapy. Another clinically relevant scenario for MRD implementation was the use of MRD negativity to support against escalation of systemic therapy in *EGFR*-mutant advanced NSCLC, specifically, deferring intensification to combinations such as amivantamab plus lazertinib or osimertinib with platinum-based chemotherapy [19,20]. This illustrates the potential of MRD-guided risk stratification to individualize systemic treatment intensity in metastatic disease based on residual molecular disease status. Although being only hypothesis-generating, considering the study limitations, this real-world practice is concordant with emerging evidence that longitudinal ctDNA dynamics can inform adaptive de-escalation strategies in selected patients with advanced NSCLC, and with prospective efforts evaluating ctDNA-guided treatment continuation versus discontinuation paradigms [21,22,23]. Conversely, MRD positivity in our cohort was associated with treatment and surveillance intensification. These actions are biologically and clinically consistent with published data showing that post-treatment ctDNA MRD positivity identifies patients at high near-term relapse risk and can precede radiologic recurrence by several months, potentially creating a window for earlier intervention and closer monitoring [24,25]. Taken together, these findings reinforce the concept that MRD may move beyond prognostication toward treatment selection, provided the approach is validated in interventional trials. Importantly, prospective interventional studies are underway to define MRD-adapted escalation and de-escalation strategies in resected NSCLC [26,27,28].

Despite high specificity, MRD sensitivity was modest. This limitation is clinically relevant, as false-negative results could lead to inappropriate reassurance and premature de-escalation. The 50% sensitivity observed in our cohort is consistent with published meta-analyses reporting pooled sensitivities of 40–65% for ctDNA-based MRD detection at landmark time points in NSCLC [15,16,17]. Importantly, MRD negativity should not be equated with cure or used as a sole basis for withholding indicated therapy. The false-negative rate may reflect low tumor-shedding phenotypes, early sampling before sufficient ctDNA accumulation, infrequent longitudinal testing, and biological sanctuary sites such as the CNS. In our series, a subset of false-negative cases involved isolated CNS relapse, which is consistent with the known constraint of plasma ctDNA detection in intracranial-only disease due to reduced shedding across the blood–brain barrier (BBB) [29,30]. This observation has two practical implications: first, a MRD-negative status should not be interpreted as excluding a recurrence risk, particularly in patients at higher risk of CNS relapse; second, MRD-guided algorithms should incorporate site-specific considerations and should not supplant guideline-concordant imaging and symptom-directed evaluation.

In addition, the limited molecular lead time observed in our cohort (median of 1.3 months [range, 0.5–5.5 months] among evaluable true-positive cases) likely reflects heterogeneous and relatively infrequent sampling in real-world practice. Prospective studies with protocolized longitudinal draws have reported longer lead times, underscoring that the clinical utility of MRD surveillance is highly dependent on sampling frequency and timing [3,10,12].

To further contextualize how MRD is applied in practice, we explored clinicopathologic correlates of any MRD-driven impact on management. In univariable models, tumor grade and the presence of STAS were the only factors associated with an increased likelihood of management modification, suggesting that clinicians may be more inclined to act on MRD results in the setting of established high-risk pathologic features [31,32]. However, these associations were not retained in the multivariable model, with wide confidence intervals indicating limited precision. The multivariable model included only four covariates among 32 evaluable patients, yielding fewer than eight events per variable, which is well below the recommended minimum of 10–20 events per predictor for stable logistic regression estimates. Accordingly, these findings should be viewed as hypothesis-generating and support a prospective evaluation of whether baseline pathologic risk modifies the clinical utility of MRD-guided strategies.

Our results should be interpreted within several limitations inherent to retrospective real-world analyses. The sample size and duration of follow-up were modest, and disease stages, treatment regimens, and MRD sampling schedules were heterogeneous and physician-directed, introducing confounding by indication. In addition, MRD testing was performed using both tumor-informed and tumor-agnostic platforms, and this study was not designed or powered to compare assay performance. Pooling results from a tumor-informed assay (Signatera™ (Exome)) and a tumor-agnostic assay (Guardant Reveal™), which differ in analytical methodology and sensitivity profiles, introduced additional heterogeneity. Observed differences in MRD-driven impact rates between assays (56.0% for Signatera™ (Exome) vs. 66.7% for Guardant Reveal™) should not be interpreted as reflecting differential clinical utility, given the small subgroup sizes and potential confounding by patient selection. These constraints preclude causal inference regarding the benefit of MRD-guided de-escalation or escalation strategies.

Nevertheless, this study provides clinically relevant insight into how MRD testing is being implemented outside of trials and how it is already shaping management decisions. The high specificity and PPV of MRD support its role as a marker of high relapse risk that could justify earlier or intensified evaluation and consideration of therapeutic escalation, while the observed de-escalation practices highlight the promise of MRD-guided decision-making in the absence of standardized protocols. Prospective interventional trials with prespecified sampling schedules and decision algorithms are now essential to define the optimal schedule, minimum threshold for sensitivity and specificity, and whether MRD-guided escalation or de-escalation improves patient outcomes compared with current stage-based paradigms, as well as treatment cost-effectiveness [26,27,28].

## 5. Conclusions

In this two-center real-world cohort of resected NSCLC, MRD testing was feasible, frequently altered clinical management, and demonstrated high specificity for recurrence. However, sensitivity was limited, including false-negative scenarios such as isolated CNS relapse, emphasizing the need for cautious interpretation of MRD negativity and standardized surveillance strategies. These findings support continued investigation of MRD-guided strategies in prospective clinical trials but should not be used to justify changes to standard-of-care treatment outside of such trials.

## Figures and Tables

**Table 1:** Baseline patient, tumor, and treatment characteristics (n = 34).

Characteristics	Patients (n = 34)
**Patient characteristics**	
Age at diagnosis, median (range), years	66.8 (37.5–79.9)
Gender, n (%)	
Female	19 (55.9)
Male	15 (44.1)
Smoking, n (%)	
Current/past smoker	21 (61.8)
Never smoker	13 (38.2)
Smoking duration, median (range), p/y	40.0 (8.0–100.0)
**Tumor characteristics**	
Pathological stage (AJCC 8th), n (%)	
I–II	25 (73.5)
III	4 (11.8)
Distant metastases at the time of MRD1 assessment, n (%)	6 (17.6)
Lung/pleura	2 (5.9)
Brain	2 (5.9)
Bone	2 (5.9)
Pancreas	1 (2.9)
Primary tumor localization, n (%)	
RUL	10 (29.5)
LUL	8 (23.5)
RLL	8 (23.5)
LLL	4 (11.8)
RML	3 (8.8)
RUL + RLL	1 (2.9)
Histology, n (%)	
Adenocarcinoma	26 (76.5)
LCNEC	3 (8.8)
Squamous-cell carcinoma	2 (5.9)
Other	3 (8.8)
Tumor grade, n (%)	
G3	7 (20.6)
G2	6 (17.6)
G1	3 (8.8)
NA	18 (53.0)
LVI, n (%)	6 (17.6)
STAS, n (%)	6 (17.6)
AGA, n (%)	16 (47.0)
*EGFR*	7 (20.6)
*KRAS*	4 (11.8)
*cMET*	1 (2.9)
*ROS1*	1 (2.9)
*NRG1*	1 (2.9)
*BRAF*	1 (2.9)
*ALK*	1 (2.9)
PD-L1 TPS, median (range), %	0.0 (0.0–90.0)
TMB, median (range), mut/Mb	4.72 (0.00–35.00)
MSI-high, n (%)	0 (0.0)
**Treatment characteristics**	
Surgery type, n (%)	
Lobectomy	30 (88.2)
Sublobar resection	1 (2.9)
Neoadjuvant treatment, n (%)	5 (14.7)
Platinum-based CMT + ICI	5 (14.7)
Adjuvant treatment, n (%)	11 (32.4)
Platinum-based CMT	8 (23.5)
ICI	4 (11.8)
Targeted therapy	2 (5.9)
RT	1 (2.9)

Abbreviations: AGA—actionable genomic alterations; AJCC, 8th—American Joint Committee on Cancer staging system, 8th edition; *ALK*—anaplastic lymphoma kinase; *BRAF*—v-Raf murine sarcoma viral oncogene homolog B; *cMET*—tyrosine-protein kinase Met/hepatocyte growth factor receptor; CMT—chemotherapy; *EGFR*—epidermal growth factor receptor; G—tumor grade; ICI—immune check-point inhibitors; *KRAS*—Kirsten Rat Sarcoma viral oncogene homolog; LCNEC—large-cell neuroendocrine lung carcinoma; LLL—left lower lobe; LUL—left upper lobe; LVI—lymphovascular invasion; MSI—microsatellite instability; mut/Mb—mutations per megabase; NA—not available/not applicable; *NRG1*—Neuregulin 1; PD-L1—programmed-death ligand 1; p/y—pack/years; RLL—right lower lobe; RML—right middle lobe; *ROS1*—c-Ros oncogene 1; RT—radiotherapy; RUL—right upper lobe; STAS—spread through air spaces; TMB—tumor mutational burden; TPS—tumor proportion score.

**Table 2:** Impact of MRD results on the decision-making process (n = 34).

MRD-Driven Management Impact	Patients (n = 34)
Positive impact (altered management), n (%)	20 (58.8)
Intensified radiological follow-up, n (%)	3 (8.8)
Treatment escalation, n (%)	2 (5.9)
Treatment de-escalation, n (%)	15 (44.1)

Abbreviations: MRD—minimal residual disease.

**Table 3:** MRD diagnostic performance in correlation with radiological disease recurrence/disease progression (n = 32; two cases without an evaluable MRD result were excluded from the analysis).

Diagnostic MRD Performance	Description	Patients (n = 32)
True positive, n (%)	MRD+, and disease recurred/progressed	5 (15.6%)
True negative, n (%)	MRD–, and no recurrence/progression	21 (65.7%)
False positive, n (%)	MRD+, and no recurrence/progression	1 (3.1%)
False negative, n (%)	MRD−, and disease recurred/progressed	5 (15.6%)
Sensitivity, %	TP/(TP + FN)	50.0%
Specificity, %	TN/(TN + FP)	95.5%
PPV, %	TP/(TP + FP)	83.3%
NPV, %	TN/(TN + FN)	80.8%
Overall accuracy, %	(TP + TN)/n	81.3%

Abbreviations: FN—false negative; FP—false positive; MRD—minimal residual disease; NPV—negative predictive value; PPV—positive predictive value; TN—true negative; TP—true positive.

**Table 4:** Univariable and multivariable analyses of factors associated with positive MRD-driven management impact (n = 32). Statistically significant values are shown in bold.

Factor	HR (95% CI)	*p* Value
**Univariable analysis**		
Age at diagnosis (per 1-year increase)	1.04 (0.96–1.12)	0.350
Gender	2.44 (0.55–10.83)	0.291
Smoking history	1.67 (0.37–7.42)	0.703
Primary tumor localization (right-sided vs. left-sided)	2.33 (0.53–10.27)	0.288
Primary tumor localization (upper/mid lobes vs. lower lobes)	1.00 (0.24–4.18)	1.000
Clinical disease stage (AJCC 8th, IV vs. I–III)	8.87 (0.45–176.31)	0.130
Pathological disease stage (AJCC 8th, III vs. I–II)	0.64 (0.11–3.91)	0.669
Histology (sq-cell carcinoma vs. adenocarcinoma)	4.26 (0.18–98.07)	0.492
Histology (other histology vs. adenocarcinoma)	4.23 (0.43–41.88)	0.358
Tumor grade (G3 vs. G1/2)	**48.00 (2.47–932.90)**	**0.009**
LVI (pos vs. neg)	0.11 (0.01–1.34)	0.118
STAS (pos vs. neg)	**15.89 (0.69–365.16)**	**0.044**
AGA (pos vs. neg)	0.56 (0.13–2.46)	0.477
PD-L1 TPS (≥1 vs. 0%)	1.75 (0.38–8.14)	0.702
TMB (≥10 vs. <10 mut/Mb)	1.64 (0.13–21.11)	1.000
Surgery	0.38 (0.02–9.05)	0.516
Neoadjuvant treatment	0.35 (0.05–2.51)	0.350
Adjuvant treatment	0.27 (0.05–1.29)	0.127
**Multivariable analysis**		
Age at diagnosis (per 1-year increase)	1.03 (0.85–1.27)	0.738
Gender	0.42 (0.01–24.50)	0.675
Tumor grade (G3 vs. G1/2)	2.30 (0.06–86.60)	0.653
STAS (pos vs. neg)	7.46 (0.17–324.09)	0.296

Abbreviations: AGA—actionable genomic alterations; AJCC, 8th—American Joint Committee on Cancer staging system, 8th edition; CI—confidence interval; G—tumor grade; HR—hazard ratio; LVI—lymphovascular invasion; PD-L1—programmed-death ligand 1; sq-cell—squamous-cell; STAS—spread through air spaces; TMB—tumor mutational burden; TPS—tumor proportion score.

## Data Availability

The datasets generated and/or analyzed during the current study are not publicly available due to privacy and ethical restrictions. However, they are available from the corresponding author on reasonable request and subject to applicable approvals and data-sharing agreements.

## Abbreviations

AGA—actionable genomic alterations; AJCC, 8th—American Joint Committee on Cancer staging system, 8th edition; *ALK*—anaplastic lymphoma kinase; BBB—blood–brain barrier; *BRAF*—v-Raf murine sarcoma viral oncogene homolog B; CI—confidence interval; *cMET*—tyrosine-protein kinase Met/hepatocyte growth factor receptor; CMT—chemotherapy; CNS—central nervous system; CRT—chemoradiotherapy; CT—computer tomography; ctDNA—circulating tumor DNA; *EGFR*—epidermal growth factor receptor; FDG-PET-CT—fluorodeoxyglucose positron emission tomography–computed tomography; FN—false negative; FP—false positive; G—tumor grade; HR—hazard ratio; ICI—immune check-point inhibitors; IQR—interquartile range; *KRAS*—Kirsten Rat Sarcoma viral oncogene homolog; LCNEC—large-cell neuroendocrine lung carcinoma; LLL—left lower lobe; LUL—left upper lobe; LVI—lymphovascular invasion; MRD—minimal residual disease; MRI—magnetic resonance imaging; MSI—microsatellite instability; mut/Mb—mutations per megabase; NA—not available/not applicable; NPV—negative predictive value; *NRG1*—Neuregulin 1; NSCLC—non-small-cell lung cancer; PD-L1—programmed-death ligand 1; PPV—positive predictive value; p/y—pack/years; RLL—right lower lobe; RML—right middle lobe; *ROS1*—c-Ros oncogene 1; RT—radiotherapy; RUL—right upper lobe; sq-cell—squamous-cell; STAS—spread through air spaces; TN—true negative; TMB—tumor mutational burden; TP—true positive; TPS—tumor proportion score.
